# Supplementary material for: Machine learning guided aptamer refinement and discovery
Source: Nat Commun. 2021 Apr 22;12:2366. doi: 10.1038/s41467-021-22555-9 (PMC8062585; doi:10.1038/s41467-021-22555-9)
Supplement: Supplementary file 1 — Supplementary Information [file 41467_2021_22555_MOESM1_ESM.pdf]

Supplemental Tables

Supplemental Table ST1: PD affinity levels and partitioning outcomes. Empty forward primer particles are excluded from the percentage calculation.

Round	Affinity level	Particles screened (millions)	Aptamer particles screened (millions)	Proportion passing threshold	Positive aptamers collected (thousands)	Negative aptamer collected (thousands)	Positive unique clusters	Negative unique clusters
1	<2 μM	80	16	0.50%	80	100	292,009	292,901
	<512 nM	80	16	0.25%	40	100	183,959	273,169
2	<2 μM	6.2	1.2	6.7%	80	100	24,492	53,976
	<512 nM	12	2.4	3.5%	85	100	19,304	37,186
	<128 nM	12	2.4	1.5%	35	100	17,861	32,859

Supplemental Table ST2: Bead stringency thresholds in read counts.

Experiment	Stringency	# of reads required to achieve 20% expected bead coverage (positive)	# of reads required to achieve 20% expected bead coverage (negative)
Original PD	< 2 uM	1373	1170
Original PD	< 512 nM	1469	440
Original PD	< 128 nM	1311	575
MLPD	< 512 nM	26	26
MLPD	< 128 nM	59	55
MLPD	< 32 nM	204	192
MLPD	< 8 nM	374	387

Supplemental Table ST3: List of aptamers with full K_D curves. Grey / white alternating bands correspond to affinity thresholds and bolded rows indicate sequences inconsistent with the MLPD affinity threshold.

Name	MLPD Affinity	Sequence Origin	ML Walked / Truncated	KD (nM)	TGGATAG on Loop<br>Yes: Completely within loop for at least 1 of top 10 structures;<br>No: Not completely in loop but may overlap;<br>N/A: Not in sequence
G13 (23nt)	<8 nM	ML	Yes	1.5	Yes
E2_w_4	<8 nM	PD	Yes	5.7	No
M1_w_4	<8 nM	ML	Yes	6.1	No
E1_w_4	<8 nM	PD	Yes	6.4	Yes
G13	<8 nM	ML	Yes	7.8	Yes
G12	<8 nM	PD	No	8.0	Yes
E3_w_4	<8 nM	PD	Yes	8.1	Yes
G_R2_2	<32 nM	PD	No	10.5	No
G12 (23nt)	<32 nM	PD	Yes	11.0	Yes
E3_w_3	<32 nM	PD	Yes	17.9	No
G_R2_1	<32 nM	PD	No	22.7	No
G_R2_3	<32 nM	PD	No	26.5	N/A
G_R2_22	<128 nM	PD	No	40.1	No
E1_s_2	<128 nM	PD	No	53.9	No
E2_s_2	<128 nM	PD	No	57.2	No
G_R2_24	<128 nM	PD	No	60.3	No
G_R2_21	<128 nM	PD	No	78.1	No
G_R2_23	<128 nM	PD	No	96.2	Yes
G_R2_17	<128 nM	PD	No	111.9	N/A

G_R2_16	<128 nM	PD	No	117.1	N/A
G_R2_19	<512 nM	PD	No	118.2	N/A
G_R2_20	<512 nM	PD	No	131.0	N/A
G_R2_15	<512 nM	PD	No	152.9	No
G_R2_14	<512 nM	PD	No	179.0	No
G_R2_12	<512 nM	PD	No	245.5	No
E3_s_1	<512 nM	PD	No	274.5	No
G_R2_13	<512 nM	PD	No	314.1	N/A
G_R2_18	>512 nM	PD	No	390.4	N/A
G_R2_9	<512 nM	PD	No	399.6	No
G_R2_8	<512 nM	PD	No	467.0	No
G_R2_10	>512 nM	PD	No	528.3	No
G_R2_11	>512 nM	PD	No	552.8	N/A
G_R2_6	>512 nM	PD	No	596.1	No
G_R2_7	>512 nM	PD	No	675.6	N/A
G_R2_5	>512 nM	PD	No	1030.0	N/A
G_R2_4	>512 nM	PD	No	1133.0	N/A

Supplemental Table ST4: Candidates proposed broken down by source & walking model

Seed Source	Original Seed Count	Random Walk	Walk with Count Model	Walk with Binned Model	Walk with Superbin Model	Total walks per seed
**Random**	177	1766	1773	1765	1764	7068
**Experimental**	400	3985	3999	3980	3992	15956
**Counts model**	4996	4993	4995	4990	4995	19973
**Binned**	4991	4983	4995	4999	4995	19972
**Superbin**	4990	4980	4993	4994	4994	19961
**Total walks per strategy**		20707	20755	20728	20740	82930

Supplemental Table ST5: Percent of seeds made better

For each seed source + walking combination, this table shows what percent of the generated sequences were better than the initial seed. Random seeds were all K_D > 512 nM. In all cases, the random_sampler_walk performs worse than walking guided by an ML model.

Seed source	Model for walking	Seed < 128 nM	128 nM > Seed < 512 nM	Seed > 512 nM
experimental	binned	1.24	7.74	9.05
experimental	counts	1.23	6.16	8.73
experimental	random	0.44	2.0	2.54
experimental	superbin	3.10	10.37	9.24
ml	binned	0.00	4.14	3.71
ml	counts	0.00	4.14	2.86
ml	random	0.00	0.68	0.24
ml	superbin	1.54	6.94	3.74
random	binned	NaN	NaN	1.42
random	counts	NaN	NaN	2.26
random	random	NaN	NaN	0.00
random	superbin	NaN	NaN	2.15

Supplemental Table ST6: Percent of seeds not made worse
This table is the same as Supplemental Table 3 (above) but looking at the percent of sequences that are not worse than their seed sequence as opposed to the percent that are better.

seed source	model for walking	seed < 128 nM	128 nM > seed < 512 nM	seed > 512 nM
experimental	binned	9.42	21.01	100.00
experimental	counts	9.70	21.37	100.00
experimental	random	3.96	6.61	100.00
experimental	superbin	11.08	20.62	100.00
ml	binned	18.75	16.55	100.00
ml	counts	3.08	22.76	100.00
ml	random	3.03	3.40	100.00
ml	superbin	10.77	18.06	100.00
random	binned	NaN	NaN	100.00
random	counts	NaN	NaN	100.00
random	random	NaN	NaN	100.00
random	superbin	NaN	NaN	100.00

Supplemental Table ST7: AUC of models on random walks from experimental seeds in the training set.

model	Counts		Bin		SuperBin	
stringency	512 nM	128 nM	512 nM	128 nM	512 nM	128 nM
type	auc	auc	auc	auc	auc	auc
dist_range						
0-2	0.747 +/- 0.024	0.692 +/- 0.031	0.736 +/- 0.024	0.738 +/- 0.026	0.758 +/- 0.023	0.742 +/- 0.026
2-4	0.698 +/- 0.009	0.65 +/- 0.012	0.694 +/- 0.009	0.702 +/- 0.011	0.706 +/- 0.008	0.747 +/- 0.01
4-20	0.669 +/- 0.006	0.64 +/- 0.009	0.66 +/- 0.006	0.673 +/- 0.008	0.671 +/- 0.006	0.698 +/- 0.008

Supplemental Table ST8: Truncation sequences with model scores and estimated affinity ranges from particle display.

	Sequence ID	Sequence	Length	Estimated K_D	Median model score	Model score variance
G12	G12	ACGTTTTTGGTGGATAGCAAATGCCAGGGCCCTTTTTTGA	40	< 8 nM	N/A	N/A
	G12.1	ACGTTTTTGGTGGATAGCAAATGCCAGGGCCCTTTTTTG	39	< 16 nM	4.132	0.009
	G12.2	ACGTTTTTGGTGGATAGCAAATGCCAGGGCCCTTT	35	< 32 nM	3.702	0.347
	G12.3	ACGTTTTTGGTGGATAGCAAATGCCAGGGCC	31	< 32 nM	3.519	0.255
	G12.4	ACGTTTTTGGTGGATAGCAAATGCCAG	27	< 16 nM	3.671	0.567
	G12.5	ACGTTTTTGGTGGATAGCAAATG	23	< 16 nM	3.441	0.672
	G12.6	GTTTTTGGTGGATAGCAAA	19	< 32 nM	3.125	1.224
	G12.7	GTTTTTGGTGGATAG	15	> 512 nM	2.799	2.241
G13	G13	CAAGAGGATTTGGTGGATAGTAAATCTTTGCCTATCCAGG	40	< 8nM	N/A	N/A
	G13.1	CAAGAGGATTTGGTGGATAGTAAATCTTTGCCTATCCAG	39	< 16 nM	3.826	0.016
	G13.2	CAAGAGGATTTGGTGGATAGTAAATCTTTGCCTAT	35	< 16 nM	3.550	0.257
	G13.3	CAAGAGGATTTGGTGGATAGTAAATCTTTGC	31	< 8 nM	3.735	0.489
	G13.4	GAGGATTTGGTGGATAGTAAATCTTTG	27	< 8 nM	3.816	0.636
	G13.5	AAGAGGATTTGGTGGATAGTAAATCTT	27	< 8 nM	3.740	0.665
	G13.6	GTGGATAGTAAATCTTTGCCTATCCAG	27	> 1024 nM	2.231	0.940
	G13.7	AGAGGATTTGGTGGATAGTAAAT	23	< 16 nM	3.808	0.848
	G13.8	GAGGATTTGGTGGATAGTAAATC	23	< 8nM	3.745	0.687
	G13.9	GATAGTAAATCTTTGCCTATCCA	23	> 1024 nM	0.219	0.213
	G13.10	TTTGGTGGATAGTAAATCTTTGC	23	> 512 nM	3.337	1.403
	G13.11	AGAGGATTTGGTGGATAGT	19	< 1024 nM	3.515	1.038
	G13.12	TTTGGTGGATAGTAA	15	< 256 nM	3.214	2.216

Supplemental Table ST9: Reads per sequencing pool (PD).

Round	K_D (nM)	Positive or Negative	Total Reads	Reads passing quality
1	512	positive	87,982,511	47,282,016
1	512	negative	110,754,864	58,774,910
1	2048	positive	82,181,847	42,600,614
1	2048	negative	135,878,956	70,298,810
2	128	positive	11,814,112	10,577,639
2	128	negative	5,268,744	4,716,420
2	512	positive	39,034,598	32,381,632
2	512	negative	9,799,946	8,829,271
2	2048	positive	56,495,476	48,563,200
2	2048	negative	49,676,887	41,560,628

Supplemental Table ST10: Complete list of primers

Name	Sequence (5' to 3')	Notes
AMS N40	AGCAGCACAGAGGTCAGATGNNNNNNNNNNNNNNNNNNNN NNNNNNNNNNNNNNNNNNNNCCTATGCGTGCTACCGTGAA	N40 single strand DNA library
AMS FP	AGCAGCACAGAGGTCAGATG	Forward primer for PCR amplification
AMS RP	TTCACGGTAGCACGCATAGG	Reverse primer for PCR amplification
AMS aminoFP	/5AmMC6//iSp18//iSp18/AGCAGCACAGAGGTCAGATG	Amino forward primer for particle conjugation
AMS FAM FPC	/56-FAM/CATCTGACCTCTGTGCTGCT	FAM primer for forward primer beads QC
AMS BioRP	/5BiosG/TTCACGGTAGCACGCATAGG	Biotinylated reverse primer for aptamer particle QC
P5 FP	AATGATACGGCGACCACCGAGATCTACACCGCGCATATGAGC AGCACAGAGGTCAGATG	Primer to add P5 adaptor
P7 In1 RP	CAAGCAGAAGACGGCATACGAGATCGTGATGGCGGAATTCTTCA CGGTAGCACGCATAGG	Primer to add P7 adaptor and Index
P7 In2 RP	CAAGCAGAAGACGGCATACGAGATACATCGGGCGGAATTCTTCA CGGTAGCACGCATAGG	Primer to add P7 adaptor and Index
P7 In3 RP	CAAGCAGAAGACGGCATACGAGATGCCTAAGGCGGAATTCTTCA CGGTAGCACGCATAGG	Primer to add P7 adaptor and Index
P7 In4 RP	CAAGCAGAAGACGGCATACGAGATTGGTCAGGCGGAATTCTTCA	Primer to add P7 adaptor and

	CGGTAGCACGCATAGG	Index
P7 In5 RP	CAAGCAGAAGACGGCATACGAGATCACTGTGGCGGAATTCTTCA CGGTAGCACGCATAGG	Primer to add P7 adaptor and Index
P7 In6 RP	CAAGCAGAAGACGGCATACGAGATATTGGCGGCGGAATTCTTCA CGGTAGCACGCATAGG	Primer to add P7 adaptor and Index
P7 In7 RP	CAAGCAGAAGACGGCATACGAGATGATCTGGGCGGAATTCTTCA CGGTAGCACGCATAGG	Primer to add P7 adaptor and Index
P7 In8 RP	CAAGCAGAAGACGGCATACGAGATTCAAGTGGCGGAATTCTTCA CGGTAGCACGCATAGG	Primer to add P7 adaptor and Index
P7 In9 RP	CAAGCAGAAGACGGCATACGAGATCTGATCGGCGGAATTCTTCA CGGTAGCACGCATAGG	Primer to add P7 adaptor and Index
P7 In10 RP	CAAGCAGAAGACGGCATACGAGATAAGCTAGGCGGAATTCTTCA CGGTAGCACGCATAGG	Primer to add P7 adaptor and Index
P7 In11 RP	CAAGCAGAAGACGGCATACGAGATGTAGCCGGCGGAATTCTTCA CGGTAGCACGCATAGG	Primer to add P7 adaptor and Index
P7 In12 RP	CAAGCAGAAGACGGCATACGAGATTACAAGGGCGGAATTCTTCA CGGTAGCACGCATAGG	Primer to add P7 adaptor and Index
P7 In13 RP	CAAGCAGAAGACGGCATACGAGATGGATGTGGCGGAATTCTTCA CGGTAGCACGCATAGG	Primer to add P7 adaptor and Index
P7 In14 RP	CAAGCAGAAGACGGCATACGAGATCGAATCGGCGGAATTCTTCA CGGTAGCACGCATAGG	Primer to add P7 adaptor and Index
Google FP	CGCGCATATGAGCAGCACAGAGGTCAGATG	Rd1 seq primer for NGS
Google RP	GGCGGAATTCTTCACGGTAGCACGCATAGG	Rd2 seq primer for NGS
Google RPC	CCTATGCGTGCTACCGTGAAGAATTCCGCC	Index Seq primer for NGS

Supplemental Figures

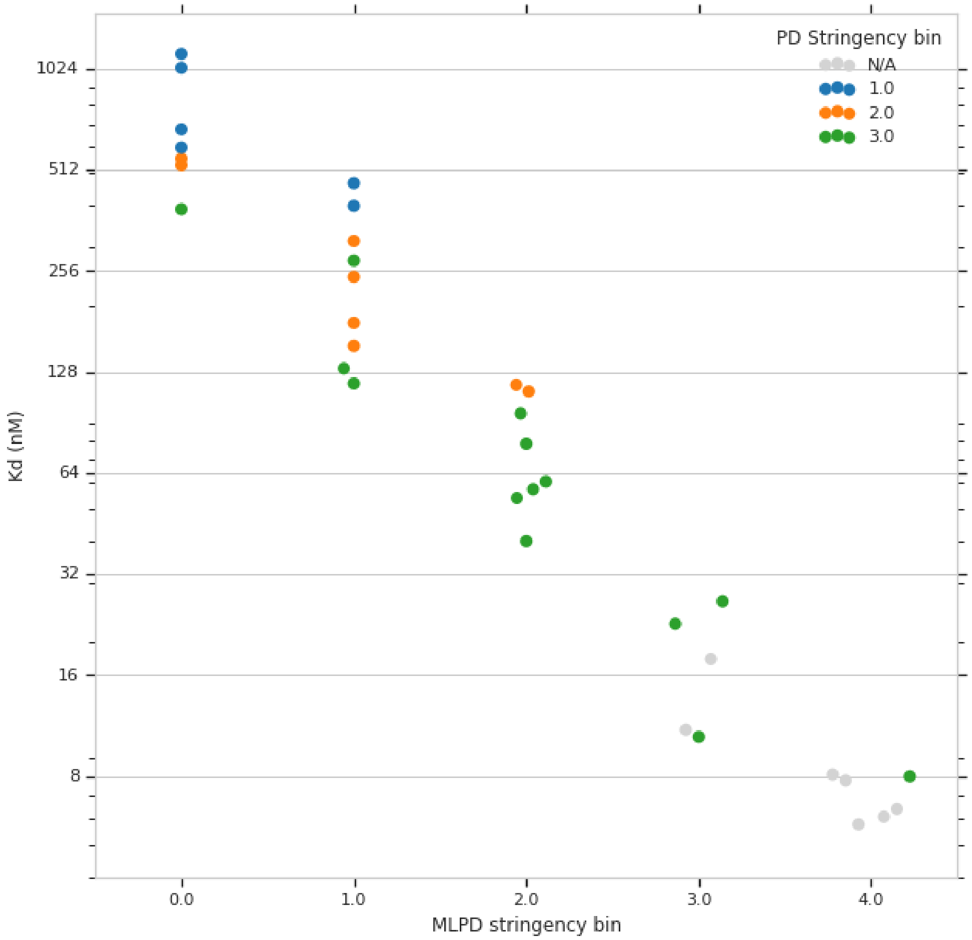

Figure S1: K_D measurement results. Experimental validation of affinity. 36 sequences were selected and full K_D curves were measured. The x-axis indicates the superbin in the MLPD validation experiment, while the color indicates the superbin in the original PD (derived sequences were not in the original PD and are colored grey). Values for each point are found in Supplemental Table 1.

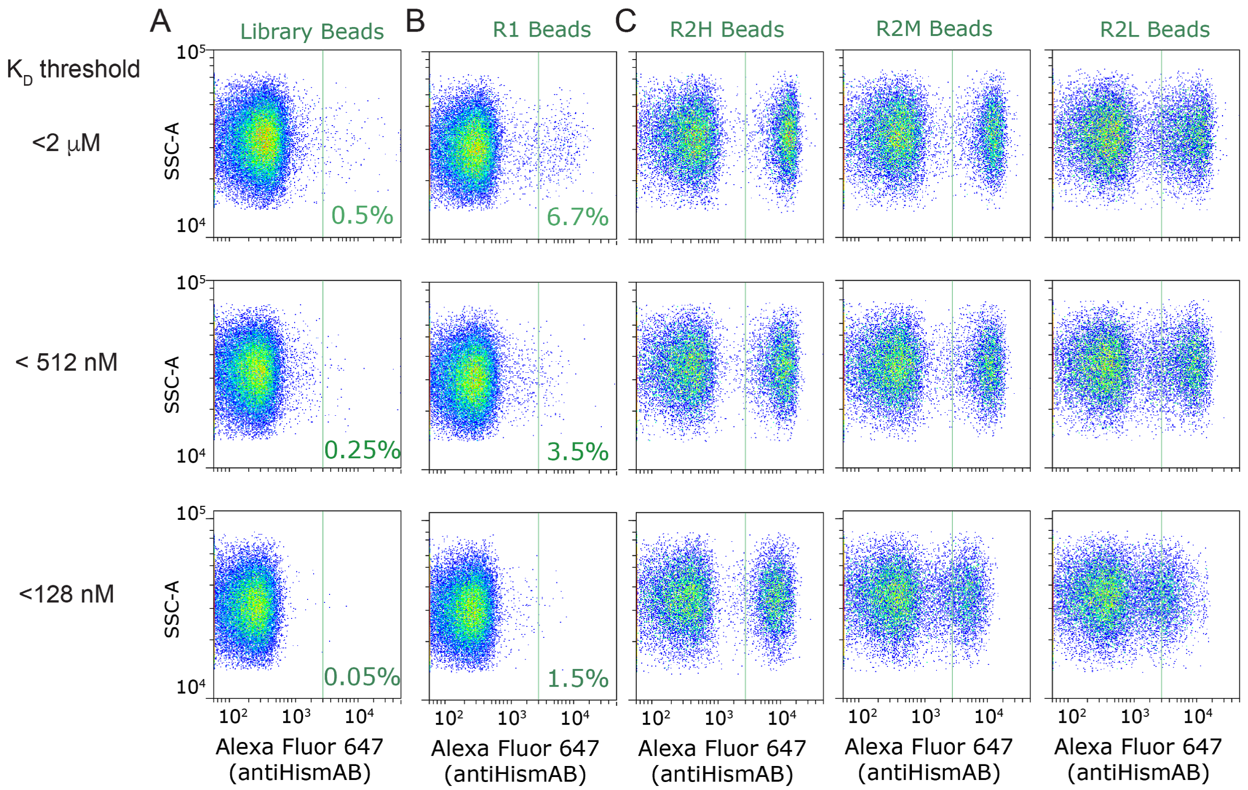

Figure S2: FACS plot from various PD rounds. FACS density plots for aptamer particles (APs) of (A) the library, the output from (B) Round 1 and (C) Round 2. PD experiments are run with increasing stringency (decreasing protein concentrations). The K_D threshold (⅓ F_{max}) shown as the green line.

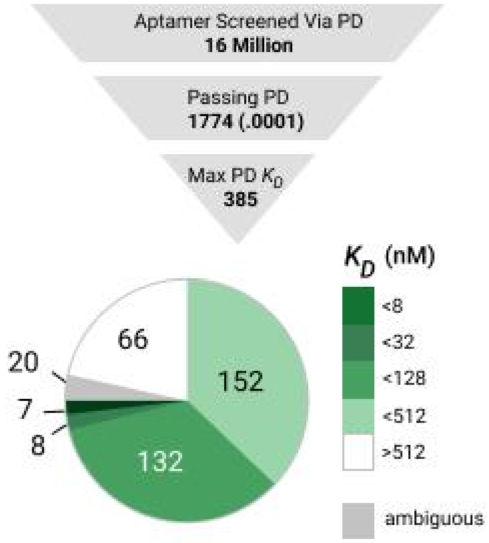

Figure S3: Performance of the 385 predicted 128 nM binders from PD in MLPD.

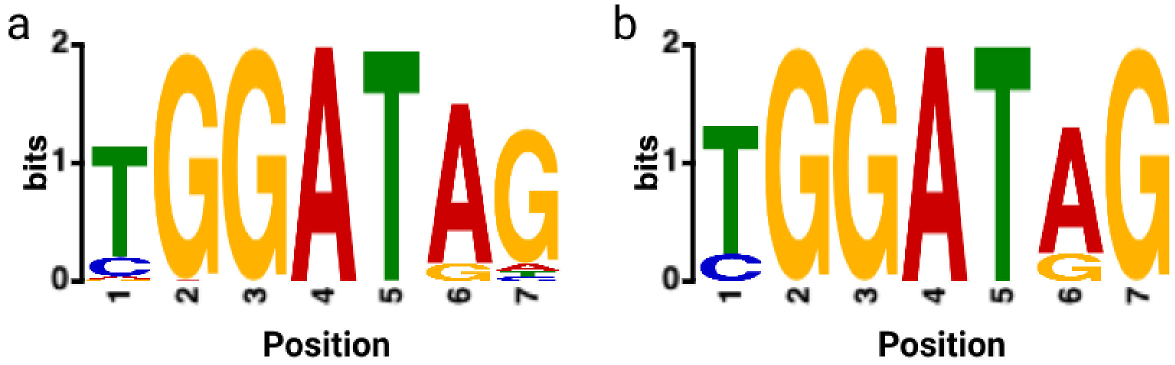

Figure S4: Motif Analysis. The highest score motif when running MEME in differential enrichment mode for: **A.)** Random sequences walked by ML models compared to their random seeds and **B.)** Sequences in the original PD test set that were observed in a positive pool vs. all sequences in the test set.

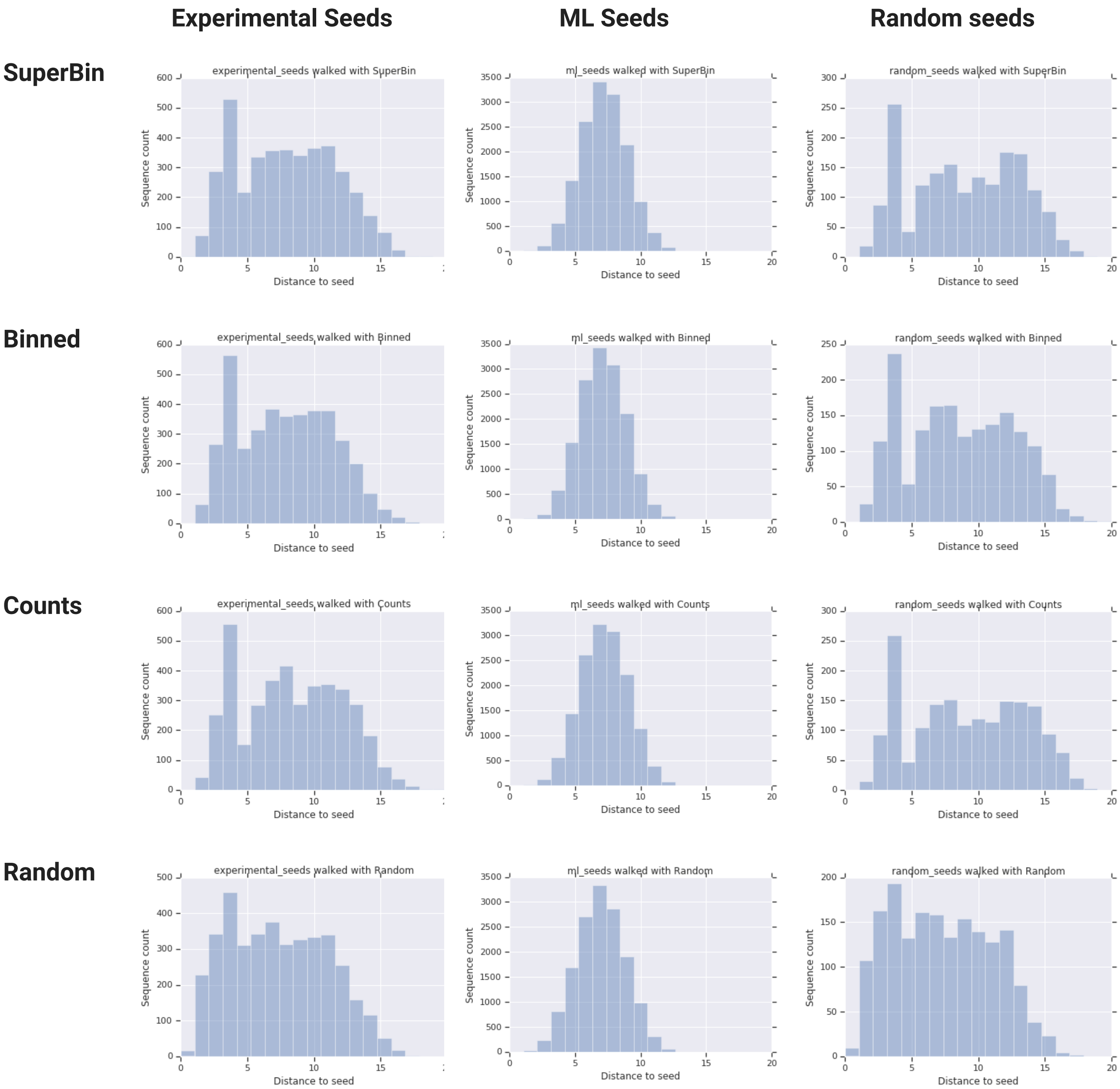

Figure S5: Distances walked from seeds. Histograms of Levenshtein distance between sequences and their seeds across different seed sets and walking models.

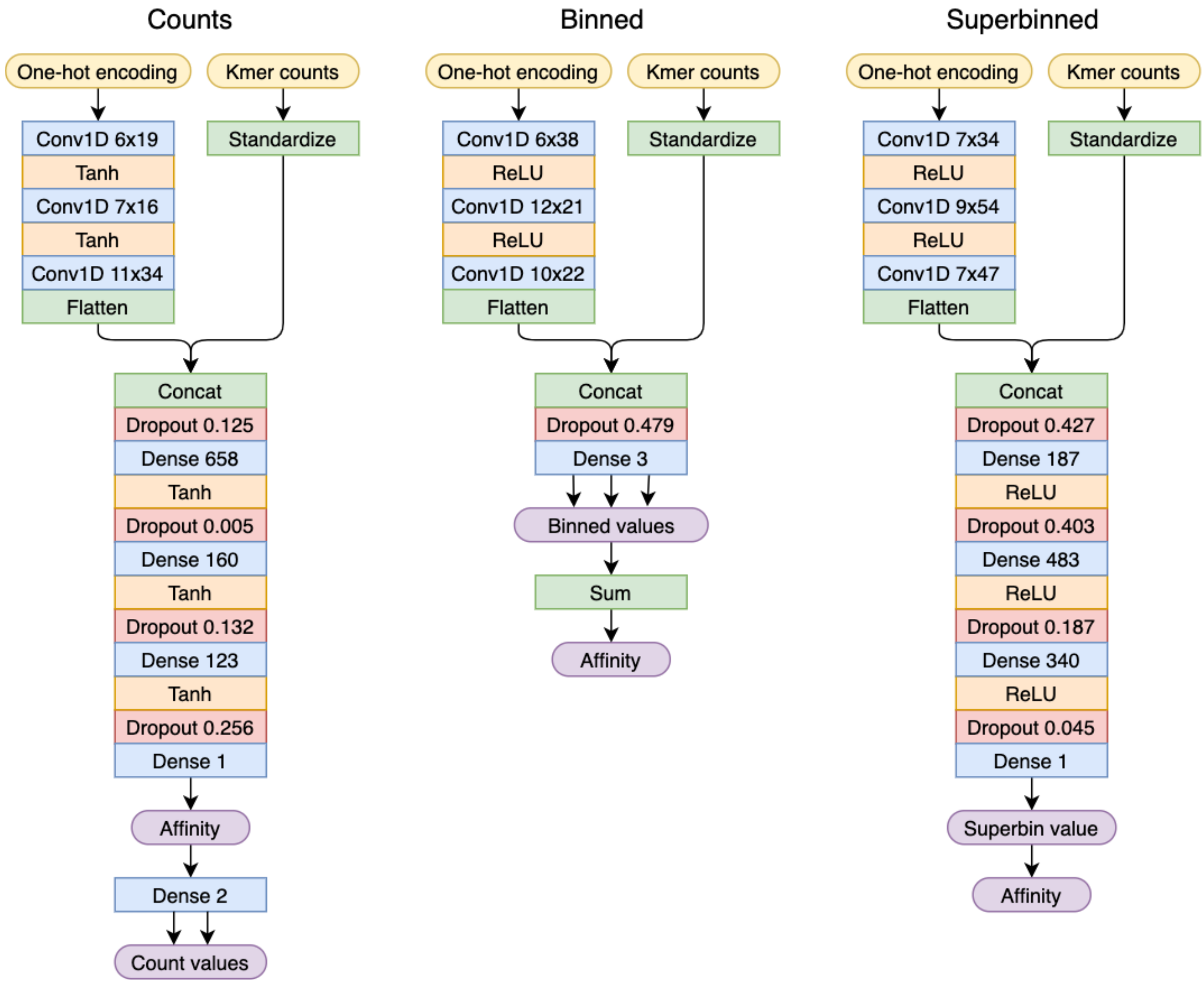

Figure S6: Detailed neural network diagrams for trained models. All numerical values shown are the results of the hyperparameter search done using grid search. Models input the one-hot encoding of the sequence and the counts of 1-4 base kmers (yellow). In each model there are three convolutional layers (blue), with the width (kernel size) and depth (number of filters) values shown. After concatenating, there are 3 additional fully connected layers with the indicated numbers of channels in the Counts and Superbinned models before the final layer reducing the size to the output size. The Counts model has the latent affinity output before the count values. For the Binned and Superbinned models, the Binned / Superbin values are the final values trained by the network. Dropout layers are only used for training, and are disabled for making model predictions.
