# Supplementary material for: Machine learning guided aptamer refinement and discovery
Source: Nat Commun. 2021 Apr 22;12:2366. doi: 10.1038/s41467-021-22555-9 (PMC8062585; doi:10.1038/s41467-021-22555-9)
Supplement: Supplementary file 3 — Reporting Summary [file 41467_2021_22555_MOESM3_ESM.pdf]

nature research

Corresponding author(s): *Double-blind peer review submissions: write DBPR and your manuscript number here instead of author names.*

Last updated by author(s): *YYYY-MM-DD*

Reporting Summary

Nature Research wishes to improve the reproducibility of the work that we publish. This form provides structure for consistency and transparency in reporting. For further information on Nature Research policies, see our Editorial Policies and the Editorial Policy Checklist.

Statistics

For all statistical analyses, confirm that the following items are present in the figure legend, table legend, main text, or Methods section.

n/a	Confirmed	
☐	☒	The exact sample size (*n*) for each experimental group/condition, given as a discrete number and unit of measurement
☒	☐	A statement on whether measurements were taken from distinct samples or whether the same sample was measured repeatedly
☒	☐	The statistical test(s) used AND whether they are one- or two-sided<br>*Only common tests should be described solely by name; describe more complex techniques in the Methods section.*
☒	☐	A description of all covariates tested
☒	☐	A description of any assumptions or corrections, such as tests of normality and adjustment for multiple comparisons
☐	☒	A full description of the statistical parameters including central tendency (e.g. means) or other basic estimates (e.g. regression coefficient) AND variation (e.g. standard deviation) or associated estimates of uncertainty (e.g. confidence intervals)
☒	☐	For null hypothesis testing, the test statistic (e.g. *F*, *t*, *r*) with confidence intervals, effect sizes, degrees of freedom and *P* value noted<br>*Give P values as exact values whenever suitable.*
☒	☐	For Bayesian analysis, information on the choice of priors and Markov chain Monte Carlo settings
☒	☐	For hierarchical and complex designs, identification of the appropriate level for tests and full reporting of outcomes
☒	☐	Estimates of effect sizes (e.g. Cohen's *d*, Pearson's *r*), indicating how they were calculated

Our web collection on statistics for biologists contains articles on many of the points above.

Software and code

Policy information about availability of computer code

Data collection	For data collection, the code used for the Flow Cytometry experiments was flowjo version 10.6.1.
Data analysis	Data analysis of the designed sequences and figure generation employed custom code. We have included this code as runnable ipython notebooks with documentation and usage information.<br><br>The code for initial data processing, model building, and walking utilize previously published approaches (e.g. ScaM for clustering) and standard model architectures/optimization strategies as described in the Methods. Note, this code depends on internal infrastructure/ libraries so it cannot be run externally, however the code has been made public at https://github.com/google-research/google-research/tree/ master/aptamers_mlpd. Note, a component of preprocessing code, the FlumeJava pipeline cannot be shared because it exposes internal APIs, however we've described the code thoroughly and provided the remaining preprocessing code, along with all the model building and walking code.

For manuscripts utilizing custom algorithms or software that are central to the research but not yet described in published literature, software must be made available to editors and reviewers. We strongly encourage code deposition in a community repository (e.g. GitHub). See the Nature Research guidelines for submitting code & software for further information.

Data

Policy information about availability of data

All manuscripts must include a data availability statement. This statement should provide the following information, where applicable:

- Accession codes, unique identifiers, or web links for publicly available datasets
- A list of figures that have associated raw data
- A description of any restrictions on data availability

-- Raw data will be published on SRA (BioProject PRJNA672779).
-- We have uploaded processed data to the editors and provided it in a public Google Cloud bucket linked from https://github.com/google-research/google-research/tree/master/aptamers_mlpd

Field-specific reporting

Please select the one below that is the best fit for your research. If you are not sure, read the appropriate sections before making your selection.

- [x] Life sciences
- [ ] Behavioural & social sciences
- [ ] Ecological, evolutionary & environmental sciences

For a reference copy of the document with all sections, see nature.com/documents/nr-reporting-summary-flat.pdf

Life sciences study design

All studies must disclose on these points even when the disclosure is negative.

Sample size	No sample size calculations were performed. For the initial library screen, the number of aptamers screened and sequenced was constrained by the throughput of the flow cytometry machine and the number of reads in the high-throughput sequencing run. In cases where we ordered specific sequences for validation, sample size was limited by the number of sequences available in the purchased library.
Data exclusions	No data were excluded from this work beyond low quality data filtered as described in Methods.
Replication	To generate candidates for machine learning training and validation, we performed three independent rounds of particle display screening. Each round, we used two/three protein target concentrations.<br><br>To validate the performance of machine learning predicated aptamer candidates, we ran full Kd measurements on 36 different candidate aptamers as independent validation of the predicted sequences and truncated sequences. For each kd measurement, we performed the Kd measurement once for each candidate aptamer with 12 concentration points in one Kd measurement.<br><br>To illustrate that we can truncate aptamer without hurting performance, we performed independent evaluations of two full length candidates, and performed Kd determinations on 29 truncated candidates. Each Kd test was repeated twice with 4 concentration points per test.
Randomization	For the ML model training, sequence clusters were put into train vs. test fold randomly. As described in the documentation, entire clusters were allocated to train/test together (ensuring the similar sequences were not spread across train/test datasets). To ensure an even distribution of cluster sizes, clusters were assigned to train/test folds in a greedy bin fashion (biggest cluster into fold0, next biggest into fold1, etc).
Blinding	n/a : Aptamer selection and affinity measurements were done with a machine, no human judgment involved in group allocation. Affinity level bin levels were determined by thresholds as described in Methods.

Reporting for specific materials, systems and methods

We require information from authors about some types of materials, experimental systems and methods used in many studies. Here, indicate whether each material, system or method listed is relevant to your study. If you are not sure if a list item applies to your research, read the appropriate section before selecting a response.

Materials & experimental systems

n/a	Involved in the study	
x		Antibodies
x		Eukaryotic cell lines
x		Palaeontology and archaeology
x		Animals and other organisms
x		Human research participants
x		Clinical data
x		Dual use research of concern

Methods

n/a	Involved in the study	
x		ChIP-seq
	x	Flow cytometry
x		MRI-based neuroimaging

Flow Cytometry

Plots

Confirm that:

- [x] The axis labels state the marker and fluorochrome used (e.g. CD4-FITC).
- [x] The axis scales are clearly visible. Include numbers along axes only for bottom left plot of group (a 'group' is an analysis of identical markers).
- [x] All plots are contour plots with outliers or pseudocolor plots.
- [x] A numerical value for number of cells or percentage (with statistics) is provided.

Methodology

Sample preparation	Aptamer particles are prepared through PCR using forward primers covalently linked to magnetic particles. Aptamer particles are then labeled with fluorescent protein through binding.
Instrument	SONY SH800
Software	flowjo version 10.6.1
Cell population abundance	>80% particles are singlets
Gating strategy	Singlets of magnetic beads were gated based on FSC-A and SSC-A and used for fluorescent intensity analysis.

- [x] Tick this box to confirm that a figure exemplifying the gating strategy is provided in the Supplementary Information.
